# Mechanical de-skewing enables high-resolution imaging of thin tissue slices with a mesoSPIM light-sheet microscope

**DOI:** 10.1364/BOE.583082

**Published:** 2026-01-08

**Authors:** Steven Moreno, Sharika Mohanan, Ahmed Elnageh, Erin Boland, Lewis Williamson, Camilla Olianti, Leonardo Sacconi, Godfrey Smith, Eline Huethorst, Caroline Müllenbroich

**Affiliations:** 1 School of Physics and Astronomy, University of Glasgow, Advanced Research Centre, 11 Chapel Lane, G11 6EA Glasgow, UK; 2 School of Cardiovascular and Metabolic Health, University of Glasgow, 126 University Place, G12 8TA Glasgow, UK; 3 European Laboratory for Non-Linear Spectroscopy, Via Nello Carrara, 1, 50019 Sesto Fiorentino FI, Italy; 4 Institute of Clinical Physiology, National Research Council (IFC-CNR), Viale Gaetano Pieraccini 6, 50139 Florence, Italy; 5 sharika.mohanan@physics.ox.ac.uk; 6 caroline.muellenbroich@glasgow.ac.uk

## Abstract

Optical clearing combined with light-sheet microscopy enables high-resolution imaging of extended tissue at scale. However, standard mesoSPIM systems are optimised for intact organs and are not suited to thin tissue slices. We present an oblique compensation scanning method using obliquely mounted samples held between refractive-index-matched slides in a 3D-printed frame. This enables mechanical de-skewing during acquisition, minimising post-processing requirements. We demonstrate feasibility in fluorescent bead phantoms and rabbit heart tissue, achieving a 4.8 × reduction in processing time and a 1.5 × improvement in axial resolution ((13.15±1.36) μm to (8.72±1.80) μm) compared to conventional *z* scan. The oblique compensation acquisition method extends mesoSPIM’s utility to fragile, laterally extended tissue sections.

## 1. Introduction

Understanding the correlation between the structure and function of biological tissues requires high-resolution imaging across large spatial scales. Mesoscale light-sheet fluorescence microscopy (LSFM), particularly mesoSPIM-type systems [1,2], has emerged as a powerful modality to image intact organs and large tissue sections with cellular resolution. Combined with tissue clearing methods [3–5], LSFM has transformed research in developmental biology, neuroscience, pathology and cardiovascular research by enabling comprehensive three-dimensional (3D) reconstructions of complex tissues [6–13].

In large animal models, however, the feasibility of whole-organ clearing and imaging is fundamentally limited by processing time and mounting constraints. In cardiac biology, where model systems aim to replicate human myocardial architecture and pathophysiology, rodent hearts often lack sufficient physiological relevance. However, the rabbit has a comparable electrophysiology to that of humans [14] and is therefore increasingly employed. Yet, due to the prolonged duration and limited penetration efficiency of clearing protocols like CLARITY, it is often necessary to isolate and image discrete regions of interest rather than the entire heart. One of the key biological applications that would benefit from mesoscale 3D imaging techniques is the investigation of cardiac remodelling following myocardial infarction (MI). Structural changes in myocardial architecture—including fibrosis, hypertrophy, and alterations in sympathetic innervation—occur over millimetre-scale regions and are not readily captured by conventional 2D histological methods. To model these processes in a translational context, the rabbit heart offers a valuable intermediate between rodent and human physiology. In particular, the percutaneous coronary artery occlusion model in rabbits closely recapitulates aspects of human MI and enables controlled induction of infarction followed by reproducible structural remodelling without the formation of epicardial adhesions [15]. This makes it an ideal system for meso-scale imaging studies aimed at resolving regional remodelling dynamics with cellular resolution. Moreover, many functional cardiovascular studies are conducted on acute cardiac tissue slices, particularly in large animal and human models, where intact organ imaging is impractical. Acute and post-fixed myocardial slices must remain within a narrow thickness range to preserve viability or structural integrity; being able to subsequently clear and image these same slices with light-sheet microscopy would provide valuable structural context to complement prior functional or electrophysiological studies, underscoring the demand for the mesoSPIM’s adaptation for mechanically unsupported, thin tissue sections.

While standard mesoSPIM imaging setups are optimised for whole-organ imaging, where specimens are embedded in a refractive index-matched medium inside a cuvette, adapting these techniques to thin cardiac slices, such as vibratome-cut sections, presents unique challenges. In contrast, inverted Single Plane Illumination Microscopy [16] and Oblique Plane Microscopy [17,18] configurations allow for direct, horizontal placement of tissue sections on a microscope stage, simplifying their imaging, albeit at the cost of tilted detection geometries that intrinsically produce skewed data sets. Imaging with such systems necessitates computational de-skewing methods [19,20] to digitally correct for spatial distortions. These approaches can introduce interpolation artifacts [21], increased processing time and higher data storage requirements. Mechanical de-skewing approaches have previously been demonstrated in oblique light-sheet systems [22]; however, it has not yet been shown whether such strategies can reliably support the imaging of fragile, laterally extended tissue slices on a mesoSPIM-type light-sheet microscope, which is inherently optimised for self-supporting intact organs and bulk cleared specimens. Acute vibratome-cut tissue slices, routinely used for functional assays in cardiac electrophysiology and neuroscience, therefore require adapted mounting and acquisition strategies on existing mesoSPIM hardware to enable multimodal, correlative imaging of tissue structure and function—an essential capability for linking physiological dynamics to underlying microanatomy in both the heart and the brain.

**Fig. 1.**
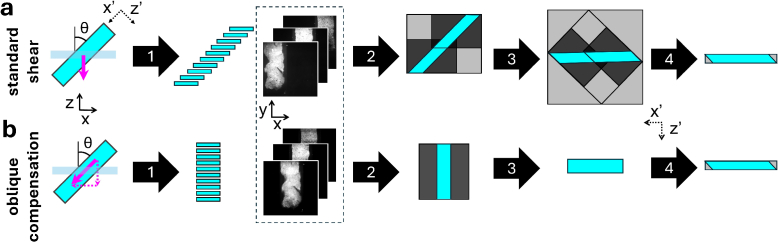
Oblique scanning geometry and volume reconstruction for the **a)** standard shear and **b** oblique compensation acquisition method. Stage scanning direction (magenta arrows) are indicated with respect to the laboratory coordinate system ( x,z ). The volume reconstruction produces a stack orientated in the sample coordinate system ( $x^{\prime },z^{\prime }$ ). Dashed inset: raw camera images, x,y plane. Standard shear acquisition method: 1) Sample acquisition produces sheared raw data stack in which the sample translates from left to right with each image. 2) For laterally extended samples, acquisition would be tiled along the $x^{\prime }$ direction. Black regions denotes empty pixels and grey regions denote padding required to perform computational deskewing. 3) Computational shearing involves rotation and additional padding. Cropping of dataset is only possible after this process (4). Oblique compensation method: 1) Sample acquisition produces raw data stack in which the sample remains in the central part of the field of view 2) Unlike the standard shear acquisition method, no tiling is required to accommodate the $x^{\prime }$ dimension. 3) Rotation of the data stack by 90degree. Pixels without content can be cropped to reduce data size. 5) Scaling along $z^{\prime }$ and shearing along $x^{\prime }$ of the data stack reconstructs the sample volume.

In this study, we introduce a custom mounting and optical de-skewing method that enables high-resolution imaging of laterally-extended cardiac tissue slices using a mesoSPIM LSFM. Cleared tissue sections are mounted between refractive index-matched quartz slides held in a 3D-printed frame [23], which is positioned at a $45^{\circ }$ angle between the illumination and detection axes. During acquisition, the sample is translated obliquely through the light sheet via sequential lateral and axial stage movements, resulting in a shear-free image stack with the specimen consistently centred within the field of view (Fig. 1). This oblique compensation acquisition method minimises data size and the need for computational de-skewing, significantly reducing processing time while improving axial resolution. We note that this method does not eliminate computational shearing entirely; it performs mechanical compensation during acquisition to pre-align the sample with its coordinate system, thereby greatly simplifying subsequent processing.

We validate this method in three key experiments. First, we compare the spatial resolution of the system across different mounting strategies. We then quantifying improvements in data processing speed and storage requirements for oblique compensation versus standard shear acquisitions. Finally, we validate the oblique compensation acquisition method by imaging a 0.5 mm-thick ventricular section from a rabbit heart, stained for nuclei and cell membranes. This approach provides a biologically translatable platform for exploring the structural basis of post-infarction structural remodelling, a process critical to understanding arrhythmogenesis and functional recovery. Importantly, retaining the three-dimensional integrity of the sample permits the extraction of volumetric data that would otherwise require hundreds of serial histological sections.

Our findings demonstrate that the oblique compensation acquisition method improves axial resolution, reduces computational overhead, and enables high-quality volumetric imaging of structurally delicate tissue sections. This approach extends the mesoSPIM’s utility beyond whole-organ imaging, providing a practical solution for studying thin, laterally extended biological specimens with minimal post-processing artifacts in cardiovascular and other biomedical research contexts.

## 2. Materials and methods

### 2.1. Sample preparation

All animal experiments were approved by the British Council for Animal Research and were conducted in accordance with the UK Animals (Scientific Procedures) Act 1986 and guidelines from Directive 2010/63/EU under Project Licence (PP5254544). All animals were kept and treated in compliance with the local regulations for animal welfare. See Supplement 1 for supporting content on detailed tissue processing steps for MI induction, slicing, clearing and staining as well as bead sample preparation protocols.

#### 2.1.1. Sample mounting

Samples were mounted with a small drop of EasyIndex Solution (LifeCanvas Technologies, EI-500-1.52) placed on a single polished 76 mm × 26 mm × 1 mm quartz slide (Portmann Instruments, UQ-1081). The cleared tissue was carefully positioned using a paintbrush to ensure it lay flat without trapped air bubbles. A second drop of EasyIndex Solution was applied atop the tissue, followed by a second quartz slide. The sandwiched tissue was then carefully inserted into a 3D-printed slide holder. Once fully positioned, fast curing silicone (Picodent, PIC. 13007100) was injected along the edges of the mount viewing window to form a watertight seal. A custom 3D-printed topper suspended the mount from the XYZθ sample stage using no adhesives or bolts to reduce strain on the quartz glass. A syringe was used to fill the remaining volume with EasyIndex solution. The topper incorporated a syringe port and an air exhaust port to facilitate simultaneous injection of solution and expulsion of air, preventing pressure build-ups. A lid was then inserted over the injection and exhaust ports to prevent contamination by dust. The lid contained a 5 mm diameter cylindrical top, allowing it to be securely mounted on a standard 0.5-inch post holder (e.g., Thorlabs PH100/M), which was attached to the XYZθ sample stage. The outer cuvette (Portmann Instruments, UQ-1083) had dimensions of 70 × 65 × 70 mm, larger than a typical mesoSPIM cuvette to accommodate the oblique movement of our mount. The front facets of the mount measured 30 mm in width, requiring a minimum diagonal length of 2 × 30 mm within the cuvette to ensure clearance. The outer cuvette was filled with 68% TDE solution (Sigma-Aldrich, 88561) to match the refractive index of the quartz glass and EasyIndex Solution (1.46).

### 2.2. Imaging

#### 2.2.1. Mesoscale light-sheet microscope

The custom-built light-sheet microscope follows the benchtop mesoSPIM design [2]. Briefly, a laser engine (Omicron LightHub Ultra) enables selection of excitation wavelengths (Luxx CW diode laser, 405 nm, 120 mW; LuxX CW diode laser, 488 nm, 100 mW; OBIS diode-pumped solid state laser, 532 nm, 150 mW; LuxX CW diode laser, 647 nm, 140 mW) with fibre-coupled output. The laser beam undergoes expansion before passing through an electrotuneable lens (ETL, Optotune, EL-16-40-TC-VIS-5D-1-C) for axial scanning of the light sheet [24]. The ETL aperture is imaged onto a galvo scanner (Thorlabs, GVS211), which generates the light sheet. The scanner is positioned at the back focal plane of a modified Nikon camera lens (AF-S 50 mm f/1.4 G), acting as an excitation objective with an f-theta lens configuration. The galvo scanner has a beam diameter of 10 mm, corresponding to an excitation numerical aperture (NA) of 0.1. Fluorescence emission is collected by a 5× and 2× objective (Mitutoyo, 59-876, f=40 mm, NA=0.14 and 59-875, f=100 mm, NA=0.055) and imaged by a tube lens ( f=200 mm, Mitutoyo, 54-774) onto the sensor of a large-format scientific complementary metal–oxide–semiconductor (sCMOS) camera (Teledyne Photometrics, 01-Kinetix-M-C). Fluorescence is selectively detected with bandpass and longpass filters (Semrock BrightLine FF01-445/45-25, FF01-514/30-25, FF01-575/59-25 and Chroma ET655LP). The camera has a 29 mm diagonal sensor size, providing a field of view of 5.8 mm. The ETL-driven axial scan is synchronized with the rolling shutter mode of the sCMOS camera. The sample is suspended from an XYZθ stage (Physik Instrumente, 2x L-509.20DG10, 1x L-509.40DG10, ± 100 nm unidirectional repeatability 1x M-060.DG, ± 25 μ rad unidirectional repeatability) for volumetric image acquisition and the detection focus is actuated by a precision translation stage (Physik Instrumente, M-406.4PD, ± 100 nm unidirectional repeatability).

#### 2.2.2. Image acquisition

The image stack of beads in the rectangular cuvette is obtained by translating the sample in steps of 1 μ m in the z direction. In the standard shear acquisition, the sample is translated in z with a step size of 1 μ m. In the oblique compensation acquisition method, the sample is stepped in x and z with a step size of 1 μ m for an oblique scan in the $x^{\prime }$ direction. The tissue slices were imaged in the oblique scanning geometry implementing mechanical de-shearing with a z -step size of 3 μ m.

#### 2.2.3. Software and image analysis

Acquisitions were performed using the standard mesoSPIM control software, with lightweight extensions enabling the oblique compensation mode. Two additional scan modes (+ $45^{\circ }$ and − $45^{\circ }$ ) were implemented by adding optional x – z coupled stage motion, corresponding GUI toggles, and metadata flags; all functionality remains fully backward-compatible with existing mesoSPIM installations. The modified control scripts and documentation are available [25].

For volume reconstruction, the affine transformations (rotation, scaling, and shear) were implemented in Fiji using the TransformJ plugin [26]. This ensures that the processing pipeline is fully compatible with standard LSFM datasets from other microscopes and uses widely available open-source tools. The images from the standard shear aquisition only require rotation and scaling transformations, while the images from from the oblique compensation acquisition require shearing and scaling transformations. See Supplement 1 for supporting content on transform matrices.

Multi-tile datasets (10% overlap) were stitched using Imaris Stitcher, and 3D visualisation was performed using the Fiji 3DViewer plugin.

For quantitative nuclear-size measurements, we developed a Python analysis module that fits Gaussian profiles to segmented nuclei and extracts FWHM values. This code relies solely on common scientific Python libraries (Tifffile, Scikit-image, SciPy, Seaborn, Matplotlib, Pandas, NumPy) and can be downloaded [27].

## 3. Results

### 3.1. Oblique compensation acquisition for mechanical de-skewing of data sets

**Fig. 2.**
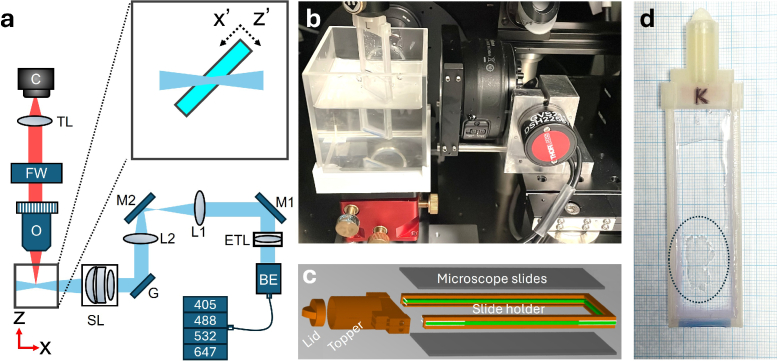
Orientation and mounting of thin tissue slices in the mesoSPIM geometry. **a)** Schematic of the mesoSPIM LSFM. BE: beam expander; ETL: electrically tunable lens, M1,M2: mirrors; L1,L2: lenses; G: galvanometric mirror; SL: scan lens; O: objective; FW: filter wheel; TL: tube lens, C: camera. Inset: orientation of the thin tissue section within the sample chamber. Laser engine provides 405 nm, 488 nm, 532 nm and 647 nm excitation wavelengths. **b)** Photograph of the mount orientation within the outer cuvette. **c)** Exploded view of computer-assisted design of the custom-made slide holder. Spacing between slides (green) corresponds to nominal thickness from vibratome cut. **d)** Photograph of mounted transmural tissue slice; epicardium on the left-hand side and endocardium on the right-hand side.

In conventional LSFM imaging, a cleared tissue section or a whole organ is placed within an inner cuvette, which is immersed in a refractive index-matching liquid inside a larger outer cuvette (Fig. 2(a)). Typically, mounted samples measure approximately 1×1×1 $cm^{3}$ . The uniform refractive index across both cuvettes ensures minimal optical aberrations as the inner cuvette is scanned through the light sheet for volumetric imaging. However, laterally extended tissue slices, for example thin vibratome-cut section, lack structural rigidity and are difficult to mount without mechanical support. To address this, we developed a custom mounting approach (Fig. 2(b-d)), where the sample is sandwiched between quartz slides held in a 3D printed frame (Fig. 2(c,d)). The slide holder maintains the correct spacing between the quartz slides that corresponds to the nominal thickness of the vibratome cut. The mount is rotated $45^{\circ }$ relative to the detection axis, resulting in an oblique scanning geometry (Fig. 2(a),inset).

In LSFMs with oblique scanning geometries, such as iSPIM or OPM, volume reconstruction typically requires computational shearing to correct for the sample’s orientation in the laboratory coordinate system (here denoted with x,y ) [21,28]. In these methods, z -scanning (Fig. 1(a)) inherently produces a sheared volume that must be computationally de-sheared. This process necessitates pixel padding of the data volume (grey areas) increasing its file size. Finally, an affine transformation, requiring additional processing time and padding, maps pixels into the correct sample coordinate system ( $x^{\prime },z^{\prime }$ ). In sheared volume reconstruction, this affine transformation involves scaling and rotation (see Supplement 1 for supporting content). Also for our laterally extended samples mounted at $45^{\circ }$ , z -scanning inherently produces a sheared volume resulting in increased data size and processing time. To address this, we introduce an optimised volume reconstruction methodology, called "oblique compensation acquisition" method, by obliquely scanning the sample through the light sheet (Fig. 2(b)).

We compensate for shear mechanically by introducing a depth-dependent lateral shift using the x -stage. The shift required for each slice in the z -stack is given by: $x_{s}=nz_{s}\tan\theta$ where θ is the angle between the sample and the detection axis, $x_{s}$ is the shift in the x -direction to re-centre the image on the camera for the n th slice and $z_{s}$ is the z step size. For our system $\theta =45^{\circ }$ and the equation simplifies to $x_{s}=z_{s}$ allowing straightforward implementation. In the oblique compensation acquisition data sets acquired in this way, the tissue slice always remains in the central part of the field of view. Once the oblique compensation acquisition dataset is acquired, a few simple steps with minimal computational overhead are applied to map pixels accurately into the correct sample coordinate system. Firstly, since the tissue occupies only the central subset of the camera frame, images are easily cropped to minimise data size. Secondly, we perform a $90^{\circ }$ rotation. This operation, reorients the dataset along the $x^{\prime }$ plane, enabling immediate visualization. Finally, scaling along $z^{\prime }$ and shearing along $x^{\prime }$ reconstructs the sample volume.

### 3.2. System characterisation

**Fig. 3.**
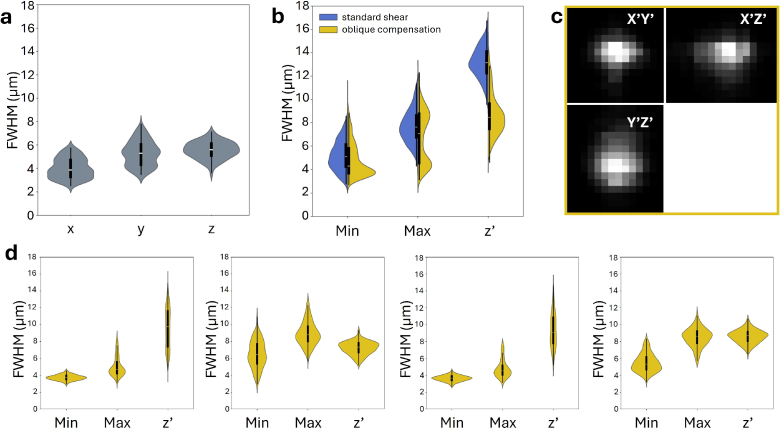
Point spread function analysis of 1 μ m diameter fluorescent beads. a) Violin plots of the full width at half maxima (FWHM) of beads ( n=196 ) embedded in agar within the rectangular cuvette. Note that these sizes are reported in the laboratory coordinate system ( x,y,z ). b) Violin plots comparing the FWHM of beads embedded in agar in sandwich mounts using the standard shear (blue, n=562 beads in 1 mount) and oblique compensation acquisition method (yellow, n=1236 beads in 4 mounts). Note, dimensions are given in the sample coordinate system ( $x^{\prime },y,z^{\prime }$ ) and lateral dimensions are reported as ${\mathrm{FWHM}}_{\min}$ and ${\mathrm{FWHM}}_{\max}$ to more robustly capture anisotropy across beads. An $R^{2}$ threshold of 0.95 ensured quality of fit. White line marks the median, black box marks the 25th and 75th percentiles. Whiskers mark out 1.5 times the inter-quartile range. c) Qualitative view of point spread function (oblique compensation acquisition) visualises astigmatism present. d) Violin plots for four different 1 mm sandwich mounts quantify variability introduced by the sample holder.

First, we quantified the spatial resolution of our LSFM employing the standard rectangular cuvette to benchmark the performance of our mesoSPIM. The violin plots (Fig. 3(a)) demonstrate a spatial resolution that is consistent with published literature for the mesoSPIM light-sheet microscope [1,2]. The mean full-width at half-maxima (FWHM) of sub-resolution fluorescent beads in the xyz directions for the standard cuvette were (4.01 ± 0.85) μ m, (5.27 ± 1.05) μ m, and (5.56 ± 0.72) μ m, respectively (number of beads, n = 196). Note that these data are reported in the conventional laboratory coordinate system ( x,y,z ) to preserve comparability with published mesoSPIM characterisations and serve solely as a performance benchmark for our mesoSPIM microscope.

We then compared the resolution achieved when mounting the sample at $45^{\circ }$ using the standard shear and oblique compensation acquisition methods (Fig. 3(b)). These datasets are analysed and reported consistently within the same sample-based coordinate system ( $x^{\prime },y,z^{\prime }$ ) ensuring a fair comparison between methods that share the same sample geometry and optical path. They are not intended to be compared directly with the bead measurements acquired in the rectangular cuvette. Because the bulk-cuvette and oblique slice-mount geometries differ fundamentally in refractive-index environment, illumination and detection propagation, and geometric distortion, expressing both datasets within a single coordinate frame would not yield meaningful or interpretable comparisons. We therefore employ two coordinate frames, each matched to the appropriate imaging geometry.

Since the relative sizes of the lateral PSFs along x′ and y can interchange across bead replicates due to slight focus adjustments, we report the lateral dimensions as ${\mathrm{FWHM}}_{\min}$ and ${\mathrm{FWHM}}_{\max}$ , a convention used by PSFJ [29] that provides a more robust measure of lateral anisotropy than assigning fixed x′ – and y -labels. The violin plots for the standard shear method (blue, left) exhibit worse resolution in all dimensions but particularly in z′ compared to the oblique compensation acquisition method (yellow, right). For the standard shear method, we obtained a mean spatial resolution in ${\mathrm{FWHM}}_{\min}$ and ${\mathrm{FWHM}}_{\max}$ and ${\mathrm{FWHM}}_{z\prime }$ directions of (5.22 ± 1.27) μ m, (7.70 ± 1.39) μ m, and (13.15 ± 1.36) μ m, respectively (n = 562 beads). For the oblique compensation acquisition method, these values were (4.88 ± 1.51) μ m, (6.82 ± 2.25) μ m, and (8.72 ± 1.80) μ m, respectively (n = 1236 beads). As expected, we observed astigmatism due to non-uniform stress on the quartz slides, leading to asymmetry in the lateral dimensions (Fig. 3(c)). The anisotropy ratio, $A:=\frac{FWHM_{min}}{FWHM_{max}}$ , is 0.72 for both modalities indicating that there is no change in the ratio of the lateral minimum to maximum bead size. We further observe a significant axial resolution enhancement in the oblique compensation acquisition method, which is evident from the lower median albeit at increased spread in the axial FWHM distribution. We attribute the 1.5× reduction ((13.15 ± 1.36) μ m reduced to (8.72 ± 1.80) μ m) in axial resolution compared to the standard shear method to the ability to fine-tune ETL parameters. The ETL sweep is confined to the sample region at the centre of the camera’s field of view, as the tissue slice remains stationary in the centre enhancing axial resolution. To assess the reproducibility of our custom 3D-printed mounts, we quantified the point-spread function across four 1 mm sandwich mounts (Fig. 3(d)). The overall resolution remained consistent across mounts, indicating robust optical alignment and mounting reproducibility. While small mount-to-mount variations were observed, primarily in the lateral dimensions, the axial ( z′ ) resolution showed minimal deviation, confirming that the sample holder design preserves the optical quality along the detection axis. This suggests that small mechanical tolerances in the 3D printing or stress in the quartz slides may lead to mild lateral astigmatism, but these effects do not significantly compromise axial resolution or imaging fidelity.

### 3.3. Computational overhead of volume reconstruction

To assess the computational efficiency of the standard shear and oblique compensation acquisition methods, we analysed the final size of the stitched data set and processing time needed for volume reconstruction. A 2×2 tiled dataset acquired with the standard shear methods (tiled in x,y ) and a 1×2 tiled dataset acquired with the oblique compensation acquisition method (tiled in y) were used to compare computational efficiency. Both datasets image the same sample volume from a cleared left ventricle slice of rabbit heart.

**Fig. 4.**
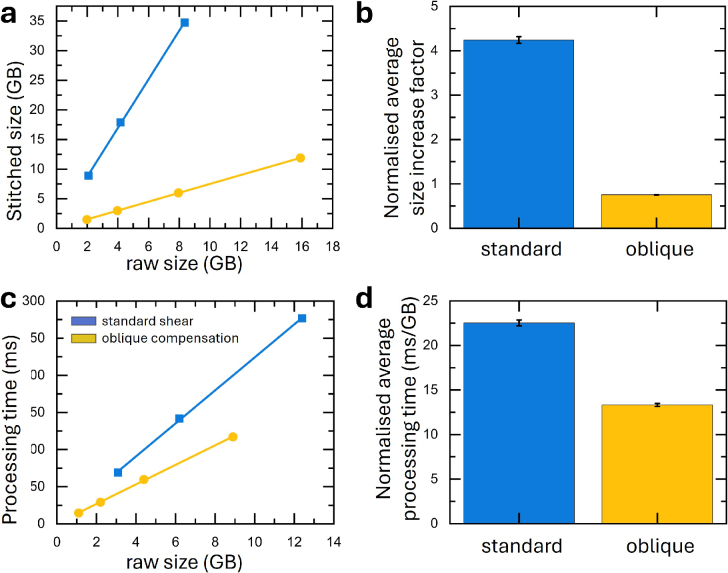
Computational overhead of volume reconstruction. **a)** Final data size after de-skewing as a function of raw file size for the standard shear (blue squares) and the oblique compensation acquisition method (yellow circles). **b)** Average data file increase. Error is standard deviation. **c)** Processing time for the affine transformation as a function of input file size for the standard shear (blue squares) and the oblique compensation acquisition method (yellow circles). **d)** Average processing time (ms/GB). Error is standard deviation.

In the standard shear method, tiling requires a 10% overlap in both the x and z dimensions requiring customisation of the tiling process from conventional methods that tile only in x and y (see Fig. 1). This redundancy in the standard shear method increases the raw dataset size and necessitates additional processing steps to merge overlapping regions. With the oblique compensation acquisition method, conventional methods of tiling in x−y can be implemented. Furthermore, the datasets obtained with the standard shear method cannot be cropped, as the skewed acquisition results in non-trivial empty spaces within the imaging volume. Consequently, the final stitched file is substantially larger than the raw dataset (Fig. 4(a)), increasing storage demands. The normalised increase in stitched file size for the oblique compensation acquisition method is by a factor of 4.24 ± 0.07 (Fig. 4(b)). Conversely, the oblique compensation acquisition method eliminates the need for tiling in the $x^{\prime }$ dimension, as the scan is performed along the oblique axis with the sample size constrained only by the working distance of the detection objective. This minimises redundancy in overlap and allows for cropping of empty pixels, further minimising the dataset size (Fig. 4(a)). In the oblique compensation acquisition method, the size of the stitched data set is actually smaller than the raw data by a factor of 0.75 ± 0.004 (Fig. 4(b)). This constitutes a reduction in stitched data size of factor 5.63 ± 0.10 of the standard shear compared to the oblique compensation acquisition method. Because the raw data in the standard shear acquisition are inherently sheared, cropping cannot be applied prior to the affine deskewing step, whereas in the oblique compensation acquisition the centred sample permits cropping before transformation; the differences in stitched volume size therefore reflect the reconstruction workflows rather than unequal fields of view.

Due to the smaller file size, computational processing time required for the affine transformation is consistently lower when using the oblique compensation acquisition method (Fig. 4(c)). The normalised processing time for the standard shear method is (22.53 ± 0.33) ms ${GB}^{-1}$ (Fig. 4(d)). Conversely, the oblique compensation acquisition method requires a normalised processing time of 13.32 ± 0.18) ms ${\mathrm{GB}}^{-1}$ . This consistent reduction by a factor of 1.69 ± 0.03 in processing time outperforms the standard shear method, highlighting the suitability of the oblique compensation acquisition method for high-throughput imaging of tissue slices, where large volumes must be processed efficiently without excessive computational burdens.

### 3.4. Laterally extended tissue imaging

**Fig. 5.**
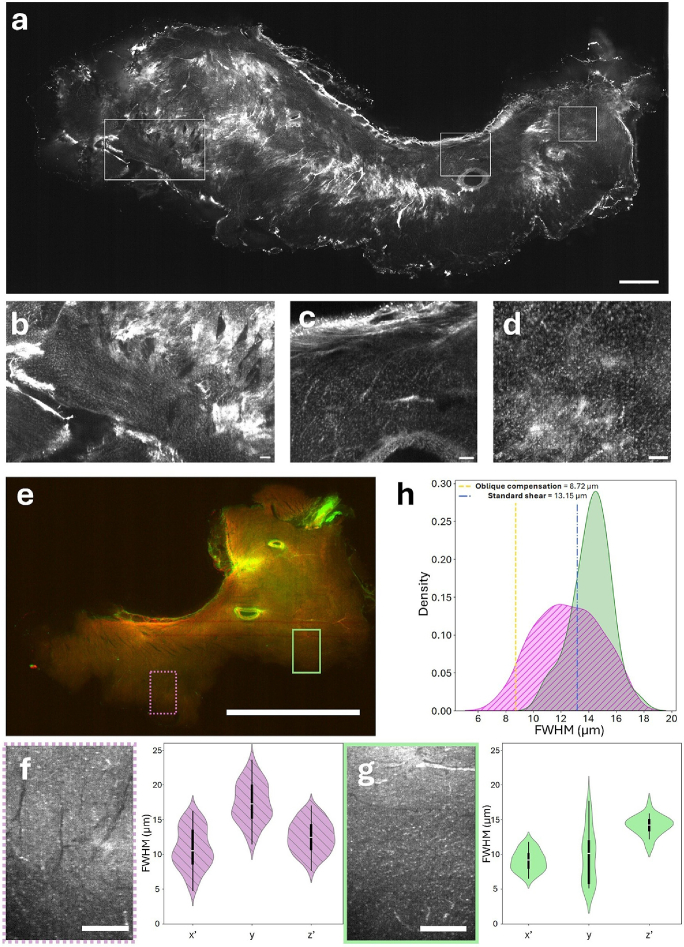
**a)** 2 mm-thick ventricular section of rabbit heart cleared with the CLARITY protocol and stained with Sytox Green. Scalebar 1 mm. **b-d)** Regions of interests, scalebar 100 μ m. **e)** 500 μ m-thick ventricular section of rabbit heart cleared with the CLARITY protocol and stained with WGA-AF488 and DAPI. **f,g)** Resolution was sufficient to analyse nuclei size across regions of interest (green and magenta ROIs, scalebar 300 μ m, n=269 nuclei). Nuclei size distribution is reported for both ROIs in the sample coordinate system ( $x^{\prime },y,z^{\prime }$ ). **h)** FWHM of the axial size of segmented nuclei in the two ROIs. Also indicated are the axial resolutions of the standard shear and oblique compensation acquisition methods as determined from the bead analysis. The enhanced axial resolution of the oblique compensation acquisition method compared to the standard shear method is essential to distinguish nuclei across the magenta ROI.

We finally demonstrate the utility of the oblique mounting and oblique compensation acquisition method by imaging a laterally-extended tissue slice. Using the oblique compensation acquisition method, we imaged a 2 mm-thick CLARITY-cleared tissue section from the left ventricle of a healthy rabbit heart (Fig. 5(a)) stained with Sytox Green to label nuclei. Resolution is sufficient to identify individual nuclei across the tissue slice as highlighted by three regions of interests (ROIs) (Fig. 5(b-d)). Next, we imaged a 0.5 mm-thick CLARITY-cleared tissue section from the left ventricle of a healthy rabbit heart (Fig. 5(e)). To visualize relevant structures, the tissue was double-stained with WGA conjugated to Alexa Fluor-488 to label cell membranes and DAPI to label nuclei (Fig. 5(a)). The oblique compensation acquisition method yields sufficient contrast and resolution to identify individual nuclei and delineate membrane boundaries throughout a 500 μ m-thick myocardial section highlighting uniform visibility of subcellular structures across the tissue depth in dense, fibrous myocardium. We identified nuclei in the DAPI channel and extracted their sizes in the sample coordinate system ( $x^{\prime },y,z^{\prime }$ ) across two representative ROIs (Fig. 5(f,g), n=269 nuclei from two ROIs in one section of one rabbit heart). Measurements of nuclear size in the oblique compensation acquisition datasets revealed dimensions consistent with the expected range for cardiomyocyte and fibroblast nuclei, typically spanning 5 μ m to 15 μ m. The nuclei were generally elliptical rather than perfectly spherical, as expected due to their intrinsic morphology and anisotropic organisation within myocardial tissue. These 3D data suggest that robust nuclei segmentation is possible due to high axial resolution and minimal distortions of the oblique compensation acquisition method. This finding opens up the possibility to quantitatively study regional heterogeneity in nuclear morphology as a biomarker in cardiac pathology. The axial FWHM of identified nuclei is plotted across the two regions of interests (Fig. 5(h)) alongside the axial resolution limits of both standard shear and oblique compensation acquisition methods. Many nuclei would not have been resolved with sufficient contrast using the standard shear method as FWHM of nuclei approaches or falls below the resolution limit. The enhanced axial resolution of the oblique compensation acquisition method enables volumetric segmentation of individual nuclei that would otherwise appear blurred or merged. The resolution improvement achievable with the oblique mounting and oblique compensation acquisition method therefore allows for the segmentation of a larger percentage of nuclei than would have been the case with the standard shear method. The nuclear size distributions observed across the two regions of interest illustrate the method’s ability to resolve and quantify subcellular morphology throughout a 500 μ m-thick myocardial slice. The improved axial resolution of the oblique compensation acquisition method ensures that even densely packed or elongated nuclei are faithfully segmented, enabling comparison of nuclear morphology between myocardial regions. This capability is particularly valuable in the study of post-MI remodelling, where nuclear hypertrophy or elongation may correlate with fibrosis, cardiomyocyte stress, or immune cell infiltration.

**Fig. 6.**
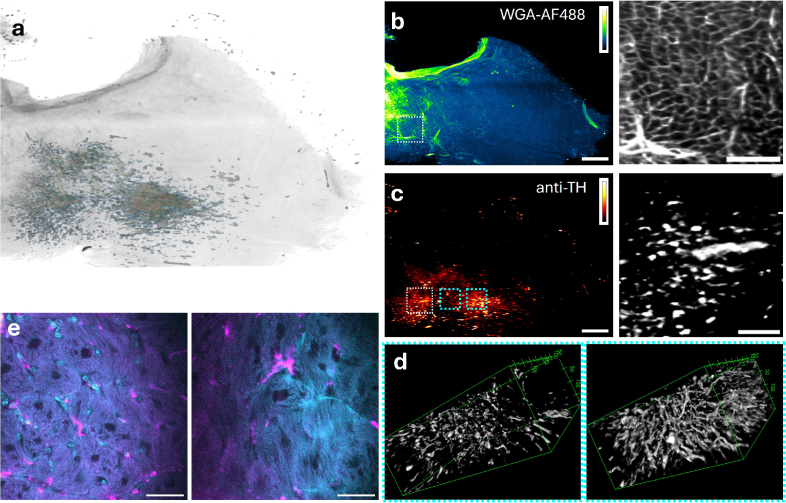
**a)** Overview of a 500 μ m-thick ventricular section of rabbit heart cleared with the CLARITY protocol and stained with **b)** WGA-AF488 and **c)** anti-TH. Scalebar 1 mm. Right hand side shows zoomed in region of interest (ROIs) indicated. Scalebar 250 μ m. **d)** Anti-TH signal spatially reconstructed in two ROIs highlighted in cyan in panel (c). **e)** High resolution multiphoton microscopy images of the sample micro-architecture. Cyan: WGA-AF488, magenta: anti-TH (scalebar 50 μ m).

As a further example, we imaged a 500 μ m-thick tissue section from the left ventricle of a rabbit heart (Fig. 6(a)) cleared with the CLARITY protocol. To visualize relevant structures, the tissue was double-stained with WGA-AlexaFluor488 ((Fig. 6(b))) to label cell membranes and immunolabelling for anti-Tyrosine Hydroxylase (anti-TH, Fig. 5(c)) to highlight sympathetic neuronal structures. The preservation of structure throughout the 500 μ m-thick myocardial slice suggests effective penetration of clearing agents, fluorescent stains and light, supported by the improved optical quality of oblique compensation acquisition imaging. Neuronal remodelling post-MI is well-documented but technically challenging to study in 3D. While light-sheet imaging has been used to study cardiac innervation patterns in whole mice hearts [30], no prior study has leveraged the mesoSPIM to map neuronal distributions in MI-induced heart slices at high resolution in rabbit heart. Our objective was to determine whether the oblique compensation acquisition method enabled 3D reconstructions of spatially continuous delicate features like sympathetic nerve fibres and nerve terminals in thick cardiac slices. We found that the innervation network could be reconstructed in selected ROIs (Fig. 6(d)) across the laterally extended tissue slice. The preservation of staining and micro-architecture was confirmed using high-resolution multiphoton microscopy, indicating that tissue integrity was maintained during the clearing and mounting process (Fig. 6(e)). Using oblique mounting and the oblique compensation acquisition method, image resolution was sufficient to distinguish innervation networks and obtain 3D reconstructions of the anti-TH-labelled structures. The antiTH-stained nerve fibres retain morphological continuity and spatial coherence, indicating the oblique compensation acquisition method’s ability to resolve extended, filamentous structures in 3D. Sympathetic innervation in the heart is known to undergo complex remodelling following MI, including regional hyperinnervation, axon sprouting, and nerve degeneration. Although this study does not yet quantify those features, our findings demonstrate that the oblique compensation acquisition method offers sufficient resolution and imaging fidelity to support future quantitative mapping of innervation architecture in post-MI tissue. The qualitative visualisation of sympathetic innervation (Fig. 6(d)) highlights the biological value of the oblique compensation acquisition method for mapping fine neuronal structures in 3D. Tyrosine hydroxylase-positive nerve fibres were consistently resolved across the thickness of the tissue section, with sufficient continuity to support spatial reconstruction. These data suggest that sympathetic axons, which are typically slender and highly branched, can be tracked over extended distances within mesoscopic volumes using our approach. While this study did not quantify innervation metrics, the imaging quality is compatible with future quantitative analysis of innervation density, fibre orientation, and regional remodelling after MI. Crucially, because the optical clearing and mounting pipeline can be applied to tissue that has undergone prior functional experiments, such as electrophysiological studies, it enables retrospective structural mapping of the same samples. This opens the possibility to directly relate functional readouts to three-dimensional structural features within the same tissue, advancing structure–function correlation in cardiac research.

## 4. Discussion

We developed a oblique compensation acquisition method for mesoSPIM-type microscopes that facilitates volumetric imaging of laterally extended tissue slices. By combining a custom, oblique mounting approach with coordinated scanning in the axial and lateral directions, this method partially compensates for the standard shear introduced by oblique sample orientation. The resulting raw data remain centered within the field of view and are easily cropped and de-sheared thereby streamlining the post-processing workflow for alignment in sample coordinates. Unlike conventional oblique scanning, which demands computationally intensive affine transformations and introduces interpolation artifacts, our approach simplifies the reconstruction pipeline by reducing the amount of padding and resampling required.

Crucially, our method differs from full mechanical de-shearing techniques reported in other systems. For example, Lin *et al.* [22] implemented mechanical shearing in a custom-built upright diSPIM-like ASLM configuration, enabling data acquisition directly into the sample coordinate system. By contrast, our method, designed for compatibility with existing mesoSPIM hardware [1,2], does not eliminate computational shearing entirely. Instead, it shifts the sample laterally during axial scanning to partially de-skew the dataset at acquisition. This reduces the volume of empty pixels, streamlines the transformation process, and decreases overall processing time by a factor of 1.69 compared to standard shear acquisitions. Additionally, at 5 × magnification, tissue is imaged along its depth in the $z^{\prime }$ direction with pixel sampling at 1.31 μ m in the x,y directions. Since the resolution of our microscope ranges from 5 μ m to 7 μ m laterally, oversampling allows pixel binning in x,y without compromising resolution.

Our system differs not only in architecture but also in its practical scope. While Lin *et al.* focus on high-NA, isotropic imaging in an upright configuration, we target a widely used platform optimised for whole-organ imaging. Our method adapts mesoSPIM for mechanically unsupported thin slices, offering a practical solution on pre-existing setups that can be adopted with minimal hardware and software modifications. Our custom mounting system supports reproducible orientation of fragile tissue slices at a $45^{\circ }$ angle. This alignment, essential for the oblique compensation acquisition, enables consistent imaging across experiments. The acquisition geometry confines the ETL sweep to the axial region actually occupied by the sample, allowing the lens to operate over a smaller and more linear portion of its response curve. Restricting the stroke in this way reduces spherical-aberration contributions during axial sweeping and improves localisation of the light-sheet waist. Additional gains arise because the confined sweep samples the beam waist in a region of minimal aberration and keeps the signal within the well-corrected central portion of the detection field. Together, these factors yield the observed two-fold improvement in axial resolution compared with the standard shear acquisition. The custom mount introduces the same intrinsic aberrations in both modes, but the standard shear acquisition amplifies their impact because the sample sweeps through more poorly corrected regions of the optical system compared to the oblique compensation acquisition method.

We also note that, for thin tissue slices, relevant image data occupy only the central portion of the Kinetix sensor. In such cases, acquisition speed could be further increased by limiting the camera readout to these central pixel lines, thereby reducing data volume and substantially accelerating acquisition. Further, we note that the lateral anisotropy observed in both acquisition modes arises primarily from the low NA and optical design of the detection objectives, which inherently limits lateral resolution and introduces mild asymmetry. This behaviour is independent of the shear-compensation strategy and therefore remains consistent between the standard shear and oblique compensation acquisitions.

One limitation of our approach is the variability introduced by the custom-made oblique tissue mounts. Stress-induced astigmatism observed in some mounts suggests that mechanical tolerances in the quartz slide holders may introduce optical aberrations, particularly in the lateral dimensions. Future iterations of the mounting system could incorporate stress-relief features or alternative materials to mitigate this effect.

Importantly, while we still apply a residual affine transformation to align data into the sample coordinate system ( $x^{\prime }yz^{\prime }$ ), the computational demands are significantly reduced. Our volume reconstruction requires only rotation and minor rescaling, in contrast to full affine transformations involving scaling, translation, and rotation in multiple axes. Our benchmarking reveals a more than fivefold reduction in stitched file size compared to standard sheared acquisition and a corresponding improvement in post-processing throughput. These savings become critical when imaging large tissue volumes or executing multi-tile acquisitions. Additionally, mechanically shifting the image reduces interpolation artefacts introduced by computational shearing. For the thin, laterally extended slices for which the oblique mounting is intended, the full lateral extent fits within the camera field of view, and stitching is therefore not required in the $x^{\prime }$ direction, reducing the potential for stitching artefacts. For larger bulk specimens that extend beyond the camera sensor, the standard mesoSPIM acquisition using the rectangular cuvette is more appropriate. Finally, along the x direction, this imaging geometry also minimises shadow and scattering artifacts [9,31].

We validated the performance of this method using 1 μ m fluorescent beads, confirming minimal introduction of detrimental aberrations like astigmatism while demonstrating substantial axial resolution improvements. Application to rabbit cardiac tissue slices demonstrated that fine-scale features such as sympathetic innervation could be reliably detected across millimetre-scale fields of view, underscoring the method’s suitability for structurally delicate tissues. Owing to its compatibility with vibratome-cut slices, this approach is readily transferable to other large-animal and human cardiac preparations including sheep, pig, and human myocardial slices.

## 5. Conclusion

We present a mechanically driven, oblique compensation acquisition imaging strategy for mesoscale light-sheet microscopy that enables high-resolution imaging of fragile, laterally extended tissue slices without requiring major hardware modifications to existing mesoSPIM systems. By eliminating the need for computational de-skewing, this method significantly reduces processing time, minimises interpolation artefacts, and improves axial resolution. Our optical de-skewing approach enhances spatial fidelity and data quality by maintaining the sample within the optimally illuminated region of the field of view during acquisition. It also reduces data volume by eliminating padding pixels, streamlining downstream analysis and storage requirements. This methodology extends the capabilities of mesoSPIM systems beyond whole-organ imaging to include vibratome-cut sections and other mechanically unsupported samples where whole organ imaging is not possible. As an accessible and scalable solution, it offers a practical upgrade path for laboratories seeking to perform high-throughput volumetric imaging with minimal post-processing overhead.

## Supplemental information

Supplement 1 Latex template for SI https://doi.org/10.6084/m9.figshare.30938759

## Data Availability

Data underlying the results presented in this paper are not publicly available at this time but may be obtained from the authors upon reasonable request.

## Supplemental document

See Supplement 1 for supporting content.
