# Supplementary material for: Mechanical de-skewing enables high-resolution imaging of thin tissue slices with a mesoSPIM light-sheet microscope
Source: Biomed Opt Express. 2026 Jan 8;17(2):671–85. doi: 10.1364/BOE.583082 (PMC12904548; doi:10.1364/BOE.583082)
Supplement: Supplementary file 1 [file boe-17-2-671-s001.pdf]

Supplemental Document

Biomedical Optics EXPRESS

Mechanical de-skewing enables high-resolution imaging of thin tissue slices with a mesoSPIM light-sheet microscope: supplement

STEVEN MORENO,[1,†] SHARIKA MOHANAN,[1,5,†] AHMED ELNAGEH,[1] ERIN BOLAND,[2] LEWIS WILLIAMSON,[1] CAMILLA OLIANTI,[3] LEONARDO SACCONI,[4] GODFREY SMITH,[2] ELINE HUETHORST,[2] AND CAROLINE MÜLLENBROICH[1,6,]

[1] *School of Physics and Astronomy, University of Glasgow, Advanced Research Centre, 11 Chapel Lane, G11 6EA Glasgow, UK*
[2] *School of Cardiovascular and Metabolic Health, University of Glasgow, 126 University Place, G12 8TA Glasgow, UK*
[3] *European Laboratory for Non-Linear Spectroscopy, Via Nello Carrara, 1, 50019 Sesto Fiorentino FI, Italy*
[4] *Institute of Clinical Physiology, National Research Council (IFC-CNR), Viale Gaetano Pieraccini 6, 50139 Florence, Italy*
[5] *sharika.mohanan@physics.ox.ac.uk*
[6] *caroline.muellenbroich@glasgow.ac.uk*
[†] *These authors contributed equally to this work.*

This supplement published with Optica Publishing Group on 8 January 2026 by The Authors under the terms of the Creative Commons Attribution 4.0 License in the format provided by the authors and unedited. Further distribution of this work must maintain attribution to the author(s) and the published article's title, journal citation, and DOI.

Supplement DOI: https://doi.org/10.6084/m9.figshare.30938759

Parent Article DOI: https://doi.org/10.1364/BOE.583082

Mechanical de-skewing enables high-resolution imaging of thin tissue slices with a mesoSPIM light-sheet microscope: supplemental document

This supplemental document gives expanded information on materials and methods necessary to reproduce the experiments detailed in the main publication.

1. MATERIALS AND METHODS

A schematic of the tissue preparation pipeline is shown in Fig. S1.

A. Tissue preparation

All animal experiments were approved by the British Council for Animal Research and were conducted in accordance with the UK Animals (Scientific Procedures) Act 1986 and guidelines from Directive 2010/63/EU under Project Licence (PP5254544). All animals were kept and treated in compliance with the local regulations for animal welfare.

A.1. Rabbit model of myocardial infarction (MI)

New Zealand White rabbits (3-4 kg) were divided in MI and Sham groups. MI was induced by permanently occluding the main left coronary artery through the percutaneous route [1]. In brief, vascular access was obtained by first dissecting the right carotid artery free, followed by insertion of a 4F sheath into the carotid artery. A 1.5 F microcatheter and 2 mm radiopaque tip were railroaded over a 0.008 ″ guide wire and directed into the left main coronary artery using a 4 F catheter under fluoroscopic imaging. Once the tip was placed in the desired location within the coronary artery, the guide wire, microcatheter and 4 F catheter were retracted. Sham animals underwent the same procedure except for placement of the tip, preventing permanent occlusion [1]. After 6-8 weeks, hearts were excised and used for Langendorff experiment.

A.2. Slicing

Following prior Langendorff perfusion protocol completion, the left anterior free wall of the myocardium was dissected from the rabbit hearts. The tissue collected from the post-MI group was taken to include healthy remote, border zone, and scar regions to enable visualisation of the transition between infarct and healthy myocardium. The tissue was submersion fixed in 4 % paraformaldehyde (PFA) and then mounted in a 10 % agarose block to support vibratome slicing (SKU: V-1000R). The tissue block was orientated to obtain transmural slices of myocardium (endocardial to epicardial) which were then transferred to PBS with 0.02 % sodium azide for storage.

A.3. Clearing

Prior to tissue transformation, cardiac slices were mounted between two microscope slides using a custom-designed 3D-printed holder. The slides were spaced to match the nominal vibratome section thickness (ranging from 0.4 mm to 2.0 mm), ensuring consistent support and structural integrity throughout processing. Tissue clearing was performed using a CLARITY-based passive clearing protocol optimised for cardiac tissue [2]. Briefly, samples were incubated at 4 °C for 72 hours with gentle agitation in a hydrogel monomer solution consisting of 4 % acrylamide, 0.05 % bis-acrylamide, and 0.25 % VA-044 initiator in 0.01 M phosphate-buffered saline (PBS). The hydrogel solution was injected directly into the sealed tissue mounts to fill the internal chamber, ensuring complete immersion and eliminating trapped air bubbles. Each mount was then placed into a sealable glass jar filled with additional hydrogel solution, which was tightly closed and stored at 4 °C for continued incubation. After 72 hours, jars were slightly opened and transferred to a vacuum degassing chamber (Jeiotech F42400-2121). Atmospheric oxygen was replaced with nitrogen gas to facilitate polymerisation, after which the jars were resealed and incubated at 37 °C for 3 hours to initiate hydrogel cross-linking. Following polymerisation, samples were removed from the jars, slide mounts and immersed in 30 mL of clearing solution comprising 200 mM

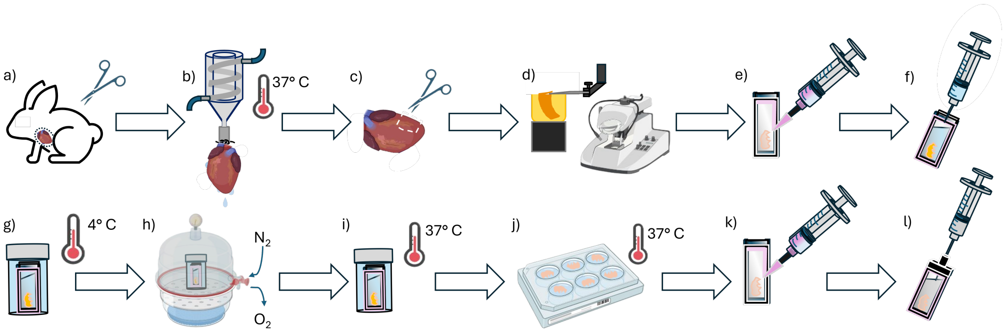

Fig. S1. Schematic of the tissue preparation pipeline. **a)** Rabbit heart excised from body, **b)** Excised hearts undergo langendorff perfusion, **c)** Left Ventricle dissected, **d)** Agar embedded LV Vibratome sliced **e)** Tissues mounted in custom watertight mount **f)** Mounts filled with hydrogel solution, **g)** Mounts refrigerated submerged in hydrogel solution, **h)** Mounts degassed with nitrogen gas, **i)** Submerged mounts incubated in nitrogen gas, **j)** Demounted tissues incubated in well plate with clearing solution, **k)** Cleared tissues re-mounted with quartz slides, **l)** Tissue mounts filled with EasyIndex™ RI matching solution.

boric acid and 4 % sodium dodecyl-sulfate (SDS) titrated to pH 8.5. Samples were incubated at 37 °C with continuous shaking prevent precipitation of SDS crystals within the tissues for approximately six months, or until optical transparency was achieved. Clearing solution was refreshed three times weekly to maintain detergent efficacy throughout the clearing period.

A.4. Staining

Immunolabelling protocols for anti Tyrosine Hydroxylase (anti-TH) and wheat germ agglutinin (WGA) have previously been described in optically cleared pig myocardium and has been applied in this study to prepared rabbit post-MI myocardial slices [3]. Cleared slices were incubated with 1:200 anti-TH (Invitrogen MA5-32984) for 3 days and washed with PBS-T 0.1x for 1 day at RT with gentle agitation followed by incubation with 1:100 WGA - Alexa Fluor 488 (Thermo Fisher, W11261) and Goat anti-mouse Alexa Fluor-647 (Invitrogen C20300) in PBS-T 0.1x for 2 days at RT with gentle agitation. The slices were then fixed with 4 % PFA in PBS for 15 min and washed in PBS for 5 min three times. Stained and fixed slices were incubated in 5 mL of EasyIndex (Life Canvas Technologies) and allowed to homogenise for 24 hours prior to imaging for complete RI matching.

A.5. Bead samples

For the rectangular sample cuvette, 1 µm diameter Dragon Green fluorescent beads (Bang Laboratories, FSDG004) were suspended in 1 % agarose (Sigma-Aldrich, A9414) at a 1:4000 dilution. The agarose solution was prepared by dissolving low-melting agarose in distilled water, heating it to 70 °C, and allowing it to cool to 40 °C before mixing with the bead suspension. The mixture was then poured into the inner cuvette, solidified at RT, and submerged in 68 % TDE (2,2'-thiodiethanol, Sigma-Aldrich, 88561) solution overnight to achieve refractive index matching [4]. For the sandwich mount, the quartz slides (Portmann Instruments, UQ-1081, 76 mm × 26 mm × 1 mm) were inserted into a 3D-printed slide holder (design files available on GitHub [?]) printed with Polylactic Acid (Verbatim # 55317 Natural 1.75mm PLA Filament printed on a Original Prusa MRK 4 3D printer with PrusaSlicer ver 2.9.2 software). Curing Silicone (Picodent®, PIC. 13007100) was applied along the edges to create a watertight seal and was left to set for 24 hours. The Dragon Green bead solution was mixed in 1 % agarose at a 1:4000 dilution and injected into the mount using a 0.4 µm diameter syringe, needle gauge 27G. The mount was filled halfway, and a mount topper was placed to seal the chamber. The solution was left to cool and gelatinise at RT for 15 minutes. Once set, the remaining space was filled with 68 % TDE solution, and the sample was left to refractive index match overnight before imaging. PSFJ [5] was used to fit PSFs to the bead images and extract lateral and axial Full width half maxima for each of the standard shear and oblique compensation acquisition datasets.

A.6. Sample alignment at 45°

Accurate orientation of the sample at 45° relative to the detection axis is critical for implementing the oblique compensation acquisition imaging approach. The alignment was performed using the following procedure. The mounted sample is first inserted into the post holder affixed to the mesoSPIM gantry holding the $XYZ\theta$ stage. The sample is raised to its upper Y-axis limit, and the mount is loosely secured using the hex lock screw, allowing rotational freedom for fine adjustment. The mount is then gently lowered into the external cuvette and translated incrementally in the Z-direction towards the rear wall until it is flush, confirming planar contact. Minor rotational adjustments are made to ensure the sample holder is parallel to the cuvette wall, avoiding excessive pressure. Once the mount is correctly positioned and laterally centred within the cuvette, the post holder is fully tightened. The sample is then rotated by 45° relative to the detection pathway using the θ stage.

B. Transform matrices

$$
\begin{aligned}
M_{\text{shear}} &= M_{\text{rotate}(y)} \cdot M_{\text{scale}(z)} \\
&= \begin{bmatrix} \cos\theta & 0 & -\sin\theta & 0 \\ 0 & 1 & 0 & 0 \\ \sin\theta & 0 & \cos\theta & 0 \\ 0 & 0 & 0 & 1 \end{bmatrix} \cdot \begin{bmatrix} 1 & 0 & 0 & 0 \\ 0 & 1 & 0 & 0 \\ 0 & 0 & \sqrt{2} & 0 \\ 0 & 0 & 0 & 1 \end{bmatrix} \\
&= \begin{bmatrix} \frac{\sqrt{2}}{2} & 0 & -1 & 0 \\ 0 & 1 & 0 & 0 \\ \frac{\sqrt{2}}{2} & 0 & 1 & 0 \\ 0 & 0 & 0 & 1 \end{bmatrix}
\end{aligned}
$$

$$
\begin{aligned}
M_{\text{no shear}} &= M_{\text{shear}(zx)} \cdot M_{\text{scale}(x,z)} \\
&= \begin{bmatrix} 1 & 0 & 0 & 0 \\ 0 & 1 & 0 & 0 \\ \frac{\cos\theta}{\sin\theta} & 0 & 1 & 0 \\ 0 & 0 & 0 & 1 \end{bmatrix} \cdot \begin{bmatrix} \frac{1}{\sqrt{2}} & 0 & 0 & 0 \\ 0 & 1 & 0 & 0 \\ 0 & 0 & \sqrt{2} & 0 \\ 0 & 0 & 0 & 1 \end{bmatrix} \\
&= \begin{bmatrix} \frac{1}{\sqrt{2}} & 0 & 0 & 0 \\ 0 & 1 & 0 & 0 \\ \frac{\sqrt{2}}{2} & 0 & \sqrt{2} & 0 \\ 0 & 0 & 0 & 1 \end{bmatrix}
\end{aligned}
$$

C. Analysis

C.1. Bead analysis

PSFJ [5] was used to fit PSFs to the bead images and extract lateral and axial Full width half maxima for each of the shear and no-shear datasets.

REFERENCES

1. M. Freeman, E. Huethorst, E. Boland, *et al.*, "A novel method for the percutaneous induction of myocardial infarction by occlusion of small coronary arteries in the rabbit," Am. J. Physiol. Circ. Physiol. **326**, H735–H751 (2024).
2. C. Olianti, F. Giardini, E. Lazzeri, *et al.*, "Optical clearing in cardiac imaging: A comparative study," Prog. Biophys. Mol. Biol. **168**, 10–17 (2022).
3. D. Vermoortele, C. Olianti, M. Amoni, *et al.*, "Precision sampling of discrete sites identified during in-vivo functional testing in the mammalian heart," Commun. Eng. **3**, 170 (2024).

4. I. Costantini, J.-P. Ghobril, A. P. Di Giovanna, *et al.*, “A versatile clearing agent for multi-modal brain imaging,” Sci. reports **5**, 9808 (2015).
5. P. Theer, C. Mongis, and M. Knop, “Psfj: know your fluorescence microscope,” Nat. methods **11**, 981–982 (2014).
